# Diagnostic reliability of O-RADS score based on non-dynamic contrast-enhanced MRI and apparent diffusion coefficient in characterization of adnexal masses

**DOI:** 10.1186/s12880-026-02498-7

**Published:** 2026-06-23

**Authors:** Basma A. Elged, Dina G. Abdelzaher, Ghada H. Abd Elraouf, Ali H. Elmokadem, Amany Hassan, Gehad A. Saleh

**Affiliations:** 1 Department of Radiology, Mansoura University, Elgomhoria St., Mansoura, 35516 Egypt; 2 Department of Pathology, Mansoura University, Mansoura, Egypt

**Keywords:** Adnexal diseases, Magnetic resonance imaging, Ovary, Ovarian neoplasm, Diffusion weighted MRI

## Abstract

**Objective:**

To evaluate the diagnostic performance and inter-rater reliability of (O-RADS) MRI based on non-dynamic contrast-enhanced (DCE) MRI and the value of apparent diffusion coefficient (ADC) in the characterization of adnexal masses.

**Methods:**

Retrospective analysis was done for 148 patients with 191 adnexal masses who underwent non-DCE MRI and diffusion-weighted imaging (DWI). Two independent radiologists classified the masses into five categories according to O-RADS-MRI and measured ADCmean values for solid and cystic components. The final diagnoses were determined by postoperative histopathology. Logistic regression analysis was performed to detect predictors of malignancy.

**Results:**

There was excellent inter-rater agreement in assessing O-RADS categories (k_w_=0.901 and 95% CI = 0.863 to 0.939). Solid tissue enhancement more than the myometrium at 30–40 s at non-DCE MRI was statistically significantly higher in malignant lesions (*p* < 0.001). There was also excellent reliability (absolute inter-rater agreement) in measuring ADCmean values. The ADCmean of solid and cystic components at cutoff values of ≤ 1.3 × 10 − 3 mm2/s and > 2.07 × 10 –3 mm²/s are perfect and excellent discriminators for differentiating malignant and benign adnexal masses (AUC = 1.000 and 0.952) respectively. Multivariate regression analysis revealed that the presence of simple fluid, low DW signal of cystic component, and ADCmean of cystic component > 2.07 × 10 –3 mm²/s were statistically significant independent predictors of malignancy in an O-RADS-MRI score > 3 adnexal lesions.

**Conclusions:**

O-RADS-MRI score utilizing non-DCE MRI is a reliable system with high PPV. ADCmean value is a non-invasive excellent discriminator for differentiating adnexal lesions that could improve O-RADS-MRI score performance and treatment plan.

**Supplementary Information:**

The online version contains supplementary material available at 10.1186/s12880-026-02498-7.

## Introduction

Adnexal masses are a common gynecologic problem, resulting in a substantial clinical workload related to diagnostic imaging and surgical management [1]. Ultrasound (US) is highly sensitive for excluding malignancy; nevertheless, 18%- 31% of adnexal masses remain indeterminate after the sonographic assessment [2, 3]. In sonographically indeterminate masses, cancer’s positive predictive value (PPV) can range from 7% to 50% [4]. MRI provides better characterization for these masses with outstanding accuracy (88–93%) for the detection of malignancy, thus decreasing unnecessary surgeries for benign lesions [5–8].

Recently, the American College of Radiology (ACR) Ovarian-Adnexal Reporting and Data System (O-RADS) MRI committee published a lexicon and risk stratification system for adnexal lesions [6]. The O-RADS-MRI stratification system is based on the previously developed ADNEX MR scoring system, which was proposed by Thomassin et al. [9]. This MRI-based score consisted of five categories according to the positive likelihood ratio (PLR) for a malignant neoplasm [10, 11]. The score utilizes dynamic contrast-enhanced (DCE)-MRI and is currently recommended to stratify the risk of sonographically indeterminate adnexal masses. However, DCE-MRI recommended by Thomassin et al. substantially expands the examination time and may be a challenge in particular patients presented with marked ascites [1, 9]. The latest updates to the O-RADS-MRI score permitted the visual analysis of enhancement patterns compared to myometrium in non-DCE-MRI. However, owing to the scarcity of data on O-RADS MRI score based on non-DCE-MRI [12], its role is still ambiguous.

The role of diffusion-weighted imaging (DWI) in the O-RADS-MRI score is based only on its qualitative analysis without considering its quantitative apparent diffusion coefficient (ADC) measurements [12, 13]. The quantitative analysis of DWI was reported to differentiate benign and malignant gynecologic masses in many studies [14–16] by displaying lower ADC values for malignant masses’ solid components [17–19]. Nevertheless, quantitative analysis of cystic components of malignant masses was reported in fewer studies [14].

Clinical implementation of the ACR RADS differs considerably among practices. The liver reporting and data system (LI-RADS) was created to standardize the reporting for hepatocellular carcinoma and has achieved outstanding approval [20]. A recent study revealed an excellent correlation between ADC and LI-RADS categories and recommended the inclusion of ADC in LI-RADS-v2018 [21]. Incorporating ADC measurements in the O-RADS-MRI score could offer a similar correlation and needs further validation.

Future directions to improve O-RADS-MRI performance mainly focus on MRI protocol and cystic component analysis [22]. Therefore, we aimed to investigate the inter-rater reliability and diagnostic performance of non-DCE-MRI and quantitative ADC measurements of both solid and cystic components for the characterization of ovarian masses using the O-RADS-MRI score.

## Methods

### Study population

**Fig. 1:**
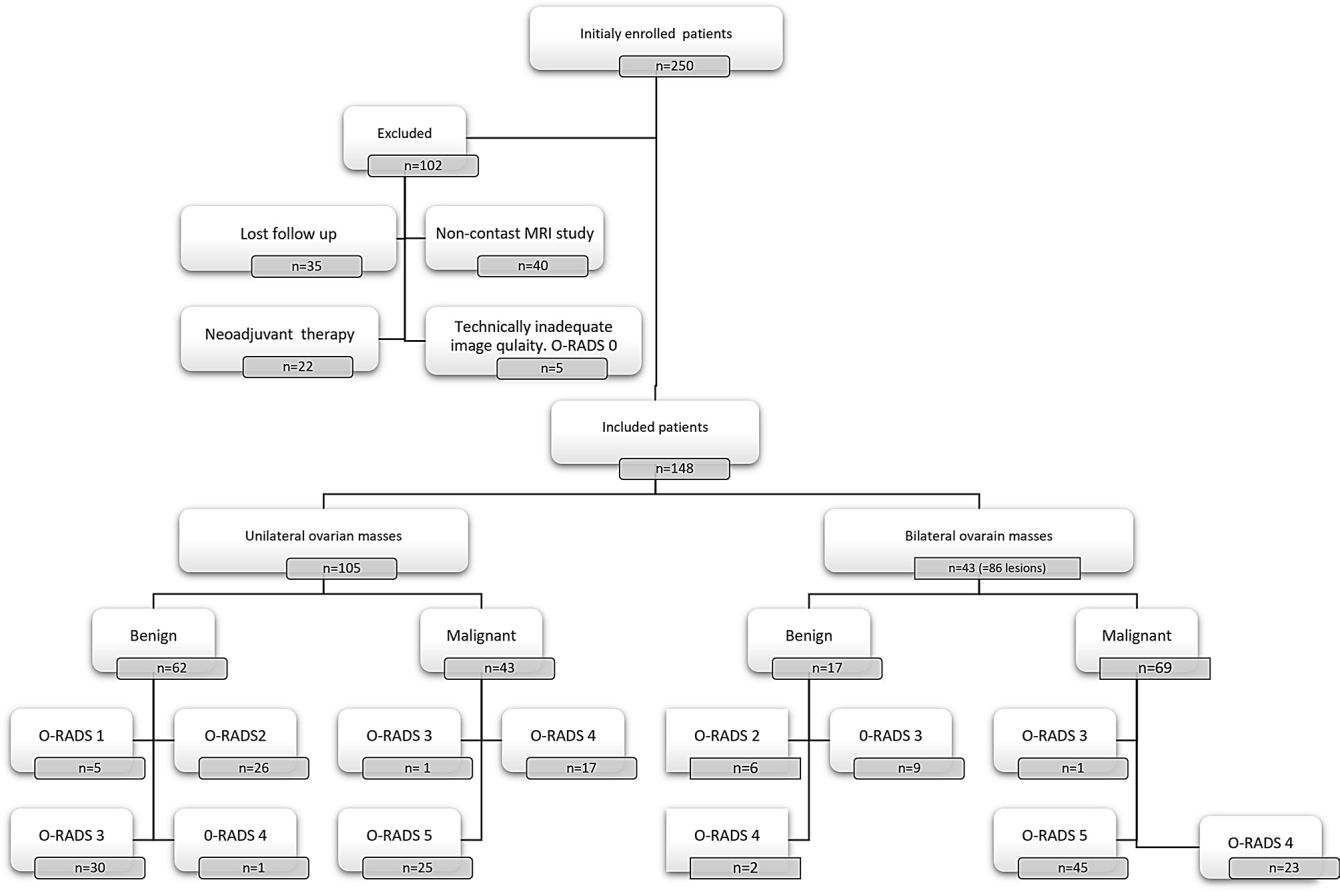
Flow chart of the study

The local institutional review board approved this retrospective study, and a waiver of consent for medical record review was received. From August 2020 to September 2022, 250 females with adnexal masses underwent pelvic MRI and DWI were enrolled. We excluded 102 patients; 35 had lost follow-up, 40 underwent non-contrast MRI, 22 received neoadjuvant chemotherapy, and the other five had technically inadequate examinations (assigned as O-RADS-MRI-0). The final study cohort consisted of 148 patients with 191 adnexal lesions. The flow chart is demonstrated in (Fig. 1).

The final diagnoses were determined by postoperative histopathology for 172 adnexal lesions in 135 patients or follow-up imaging for 19 benign lesions in 13 patients. The lesions were considered benign if they decreased in size or remained stable at 24-month follow-up imaging. The lesions were classified as benign, borderline, or malignant. Both borderline and malignant lesions were combined into one group and were compared to the benign lesions.

### MR imaging technique

#### Conventional MRI

All patients underwent pelvic MRI using a 1.5-T MR imaging scanner (Philips Ingenia, Netherlands). Patients fasted for 4–6 h; anti-peristaltic agents (hyoscine butylbromide) were injected intramuscularly to reduce bowel motion and improve image quality. All patients received a standard clinical dose of 0.1 mmol/kg (0.2 mL/kg) of the gadolinium-based contrast agent gadopentetate dimeglumine (Magnevist; Bayer Healthcare) followed by a 10 ml saline infusion. MR imaging started with high-resolution sagittal and axial T2WI (TR/TE, 3000–4000/100-110ms), FOV 250 × 300 mm, slice thickness 4 mm, interslice gap 1–3 mm and matrix 182 × 352 to 358 × 512, followed by axial T1WI with and without fat-saturation (TR/TE, 382–724/7.3–13ms.), FOV 240 × 320 mm, slice thickness 4 mm interslice gap 1–3 mm and matrix179 × 320 to 280 × 400. All patients underwent non-DCE MRI, and IV contrast (gadopentetate dimeglumine) was administered at a dose of 0.1 mL/kg with an injection rate of 2 mL/s. Post-contrast images (3D gradient echo with fat-saturation) in three planes were performed as follows; (TR/TE, 500/10ms.), FOV 260 × 216 mm, slice thickness = 3 mm, interslice gap 1 mm and matrix 263 × 171. The axial 3D acquisition started 30 s after the administration of the IV contrast agent. The non-DCE MRI protocol utilized in our study lacks time–intensity curve (TIC) acquisition or true dynamic perfusion. However, it still includes single-phase post-contrast images taken at 30–40 s for visual comparison with the myometrium.

#### DWI

DWI was performed before the contrast material injection using an axial fat-suppressed single-shot echo-planar sequence with variable b values (b = 0,500,1000 s/mm2). The diffusion imaging acquisition parameters were as follows: TR/TE = 7000/77ms, FOV = 240 × 220, matrix = 128 × 128, slice thickness = 5 mm and slice gap=1 mm.

### Image analysis

All images were transferred to a workstation (extended MR Workspace 2.6.3.5, Philips Medical Systems). Image analysis was achieved by two independent radiologists blinded to the final histopathological results (13 and 10 years of experience in gynecologic imaging).

#### Conventional MRI

Both readers classified all 191 adnexal lesions according to the five categories of ACR O-RADS-MRI Lexicon (12) (Table 1). Each reader separately recorded lesion size, laterality, type (cyst with or without solid components or solid), signal intensity on T1WI (hypointense, intermediate, or hyperintense (compared to iliopsoas muscle and fat), and signal intensity on T2WI (compared to iliopsoas muscle and cerebrospinal fluid), wall thickness, presence of septa, enhancement pattern, presence of peritoneal deposits, pathological LNs, and ascites. The degree of solid tissue enhancement in 123 lesions was compared to the outer myometrium at 30–40 s after contrast injection to decide if the lesion should be assigned an O-RADS-MRI score of 4 or 5. The lesions were assigned O-RADS-MRI score 4 if the solid tissue was enhancing less than or equal to the myometrium while lesions with solid tissue enhancing greater than the myometrium were assigned O-RADS-MRI score 5.

**Table 1:** O-RADS MRI scoring system

O-RADS MRI score	Risk category	PPV	MRI features
1	Normal ovaries	—	- No ovarian lesion. - Cyst ≤ 3 cm; either follicle, corpus luteum, or hemorrhagic cyst in a premenopausal woman.
2	Almost certainly benign	< 0.5%	- Unilocular ovarian or para-ovarian cyst with simple or endometrial fluid content (smooth wall +/- enhancement and no enhancing solid tissue). - Lesion with lipid content† and no enhancing solid tissue. - Lesion with “dark T2/dark DWI” solid tissue (homogeneously hypointense on T2 and DWI). - Dilated Fallopian tube with simple fluid contents (smooth wall/folds +/- enhancement and no enhancing solid tissue).
3	Low risk	≈ 5%	- Unilocular cyst with hemorrhagic, proteinaceous, or mucinous fluid content (smooth mural enhancement with no enhancing solid tissue). - Multilocular cyst with no lipid content and any type of fluid content (smooth septa, mural enhancement, and no enhancing solid tissue). - Lesion with solid tissue (excluding T2 dark/DWI dark): low-risk (type1) time-intensity curve on DCE MRI. - Dilated Fallopian tube; simple fluid contents with thick wall/folds or non-simple fluid with smooth wall/folds and no enhancing solid tissue.
4	Intermediate risk	≈ 50%	- Lesion with solid tissue (excluding T2 dark/DWI dark) that is enhancing ≤ myometrium at 30–40 s on non-DCE MRI. - Lesion with lipid content with large size enhancing solid tissue.
5	High risk	≈ 90%	- Lesion with solid tissue (excluding T2 dark/DWI dark) that is enhancing > myometrium at 30–40 s on non-DCE MRI. - Peritoneal, mesenteric, or omental nodularity or irregular thickening, with or without ascites.

PPV, positive predictive value (for malignancy)

Solid tissue is defined as component that enhances or a lesion having one of these morphologies: mural nodule; papillary projection; irregular septation/wall; or other larger solid portions

Sources: Thomassin-Naggara et al. [12], Pereira PN, et al. [23] and the American College of Radiology [12]

#### Qualitative DWI analysis

Both readers assessed the signal intensity of both solid and cystic components at high b-value (1000s/mm2) DWI and assigned them as low or high (compared to urine or cerebrospinal fluid). A hyperintense signal on DWI with a hypointense signal on the corresponding ADC map was considered restricted diffusion.

#### Quantitative DWI analysis

Matched ADC maps were applicable using a Phillips Advantage Windows workstation with functional tool software. Both readers separately measured ADCmean values by manually applying a two-dimensional ROI (the ROI size was ≥ 50 mm^2^) encompassing the darkest part of the solid component in 118 lesions corresponding to the highest signal at b1000 with references to T2 and contrast-enhanced images. To get accurate cutoff values differentiating between adnexal lesions without misregistration or reading bias from nearby cystic components, ADCmean solid component was not measured in 7 lesions with irregular walls and septa < 10 mm. The ADCmean of the cystic component was measured in 187 lesions by manually applying ROI encompassing purely cystic part, avoiding the lesion wall, septa, and solid tissue. ADCmean cystic component was not measured in 4 predominantly solid lesions. To ensure the reproducibility and stability of the quantitative ADC measurements, a minimum ROI of ≥ 50 mm² was maintained. This minimum area was chosen to eliminate partial volume effects from adjacent pelvic structures and to enable the reader to effectively center the ROI within the solid tissue, reducing border contamination. Seven ovarian lesions with too small solid component were excluded from quantitative sampling and were instead risk-stratified utilizing the qualitative visual assessment relative to the myometrium according to the standard ACR O-RADS MRI lexicon, instead of depending on an unstable numerical ADC cutoff. The ADCmean values were measured three times, and the measurements were averaged.

### Statistical analysis

Data were analyzed using IBM-SPSS software (version26.0) and MedCalc Statistical Software (version18.9.1). Qualitative data is N (%) compared by chi-square or Fisher’s-exact test. Quantitative data were initially tested for normality using Shapiro-Wilk’s test, with data being normally distributed if *p* > 0.050. Lesion size and ADC values was expressed as median and was compared by Mann-Whitney and Kruskal-Wallis tests, respectively. Weighted Kappa and intraclass correlation coefficient (ICC) were performed to assess the agreement between the two raters for ordinal and continuous variables, respectively. The diagnostic performance of quantitative ADC measurements was assessed by ROC curve analysis, sensitivity, specificity, PPV, NPV, and likelihood ratio (LR). Logistic regression was used to detect predictors of malignancy.

## Results

### Patients’ characteristics

The mean age (years) ± SD for patients with benign and malignant lesions was 41.9 ± 13 and 51.5 ± 13.7, respectively, and showed a statistically significant difference (*p* < 0.001) between both groups. Of 191 lesions 79(41%) were benign and 112(59%) were malignant. The most common benign and malignant adnexal masses were serous cystadenoma (26.6%) and serous carcinoma (65.1%), respectively. This relatively high prevalence of malignancy was assumed since our hospital is a tertiary care facility. The final histopathological diagnosis is illustrated in (Table 2).

**Table 2:** Final histopathological diagnosis of adnexal masses

Final histopathological diagnosis	Incidence
**Benign adnexal masses**	**60**
Serous cystadenoma	16
Serous adenofibroma	1
Mucinous cystadenoma	7
Endometriotic cyst	9
Brenner tumor	2
Fibrothecoma	6
Dermoid cyst	5
Hemorrhagic cyst	6
Tumor-like lesions: (Functional cyst, para-ovarian cyst, and hematosalpinx)	7
Subserous myoma	1
**Malignant adnexal masses**	**112**
Serous borderline tumor	14
Serous carcinoma	73
Mucinous carcinoma	6
Endometrioid carcinoma	5
Clear cell carcinoma	3
Dysgerminoma	1
Adult granulosa cell tumor	3
Secondary tumors (metastases)	7

### MRI analysis

#### Conventional MRI

The presence of simple fluid (low T1 and high T2 signal intensity) and a solid component of intermediate T2 signal intensity, solid tissue enhancement more than the myometrium at 30–40 s at non-DCE-MRI and O-RADS-MRI score > 3 was statistically significantly higher in malignant lesions. Other MRI features were illustrated in (Table 3).

**Table 3:** MRI characteristics of adnexal lesions

	Benign	Malignant	Total	*P* value
**Lesion size**	6 (4.3-9)	8 (6–10)	7 (5–10)	**0.035**
**Laterality**				**< 0.001**
**Unilateral**	62 (78.5%)	43 (38.4%)	105 (55%)	
**Bilateral**	17 (21.5%)	69 (61.6%)	86 (45%)	
**O-RADS**				**< 0.001**
**O-RADS 1–3**	76 (96.2%)	2 (1.8%)	78 (40.8%)	
**O-RADS 4–5**	3 (3.8%)	110 (98.2%)	113 (59.2%)	
**Fluid descriptors**				
Simple	58 (75.3%)	93 (85.3%)	151 (81.2%)	0.086
Lipid containing	5 (6.5%)	1 (0.9%)	6 (3.2%)	*0.083
Hemorrhagic	11 (14.1%)	20 (17.7%)	31 (16.2%)	0.508
Endometriotic	14 (17.9%)	1 (0.9%)	15(7.9%)	**< 0.001**
Proteinaceous	8 (10.3%)	14 (12.4%)	22 (11.5%)	0.65
**Solid tissue**	13(10.5)	110 (89.5%)	123 (64.4)	**< 0.001**
**Solid tissue morphologic characteristics**				**< 0.001**
Irregular walls and septae	5 (38.5%)	7 (6.4%)	12 (9.8%)	*1.000
Papillary projections	0 (0%)	13 (11.8%)	13 (10.5%)	*< 0.001
Mural nodules	0 (0%)	43 (39.1%)	43 (35%)	*< 0.001
Larger solid portion	8 (61.5%)	47 (42.7%)	55 (44.7%)	**< 0.001**
**Solid tissueT2 signal (** ***n*** ** = 123)**				*< 0.001
Low	11 (84.6%)	0 (0%)	11 (8.9%)	
Intermediate	2 (15.4%)	110(100)	112 (91.2%)	
**Solid tissue enhancement (** ***n*** ** = 123)**				*< 0.001
≤ myometrium	13(100%)	39 (35.5%)	52 (42.3%)	
> myometrium	0 (0%)	71 (64.5%)	71 (57.7%)	
**DW signal Cystic component (*n*** **= 187)**				**< 0.001**
Low	38 (50%)	109 (98.2%)	147 (78.6%)	
High	38 (50%)	2 (1.8%)	40 (21.4%)	
**DW signal solid component (*n*** **= 118)**				*< 0.001
Low	3 (37.5%)	0 (0%)	6 (5.1%)	
High	5 (62.5%)	110 (100%)	112 (94.9%)	

Lesion size data is median (Q1-Q3) and test of significance is Mann Whitney U-test. All other categorical data is N (%) and test of significance was chi-square or *Fisher’s exact test

Solid tissue was tested initially by Fisher’s exact test (Rx2) followed by creation of dummy variables to perform post-hoc multiple 2 × 2 Fisher’s exact test

There was a statistically significant difference (*P* < 0.001) between malignant and benign lesions as regards the solid tissue enhancement at non-DCE-MRI (Supplementary Fig. 1). Solid tissue enhancement ≤ myometrium was detected in 13 benign, 14 borderline, and 25 malignant lesions, while enhancement more than myometrium was detected only in malignant lesions (*n* = 71). The malignancy rates were 0%, 0%, 4.8%, 93%, and 100% for O-RADS-MRI scores of 1,2,3,4, and 5, respectively. There was a high PPV for O-RADS 4 (93%), O-RADS 5 (100%), and O-RADS 4–5 (97.3%) **(**Table 4 and supplementary Table 1).

**Table 4:** Predictive values of O-RADS in discriminating malignant from benign adnexal lesions

O-RADS	PPV	NPV
O-RADS 1	0%	39.8%
O-RADS 2	0%	29.6%
O-RADS 3	4.9%	26.7%
O-RADS 4	93.0%	51.4%
O-RADS 5	100%	65.3%
O-RADS 1–3	2.56%	2.65%
O-RADS 4–5	97.3%	97.4%

PPV: positive predictive value; NPV: negative predictive value

**Fig. 2:**
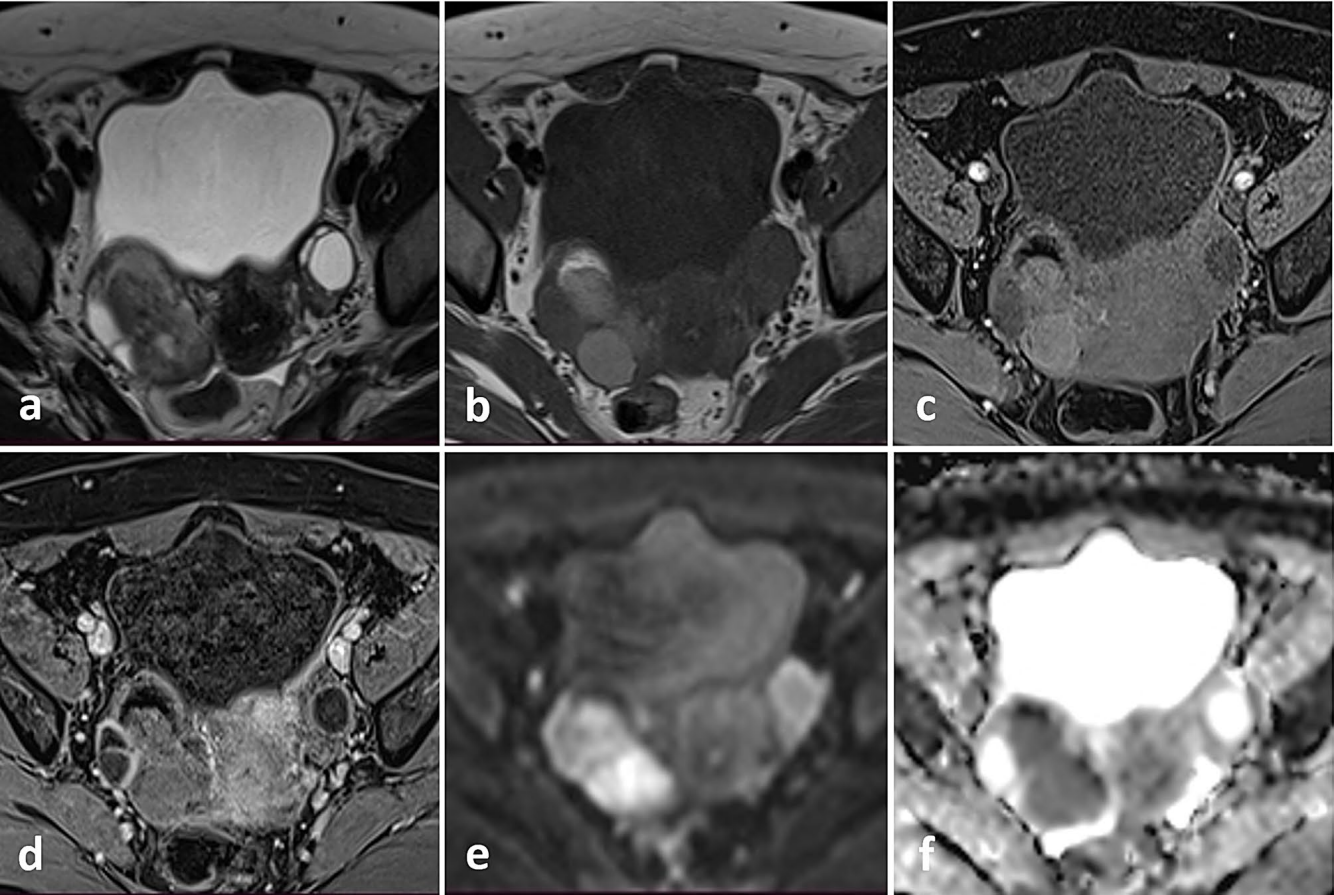
A right ovarian O-RADS MRI score 2 lesion. (**a**) Axial T2-WI shows a right-sided multiloculated adnexal mass of mixed signal intensity and left ovarian follicle (normal physiological observation). (**b**) Axial T1-WI shows high signal intensity locule at the right ovarian mass with a signal drop in T1 fat-saturated image (**c**) denoting lipid content. (**d**) Axial T1 contrast-enhanced image reveals a smooth wall, septal enhancement, and no enhanced solid tissue. (**e**) Axial DWI at b = 1000 revealed a high signal of the cystic component and hypointensity at the corresponding ADC image (**f**), denoting a restricted diffusion pattern with an ADC value of 0.907 × 10 − 3 mm2 /s. Pathology confirmed a right ovarian dermoid cyst

**Fig. 3:**
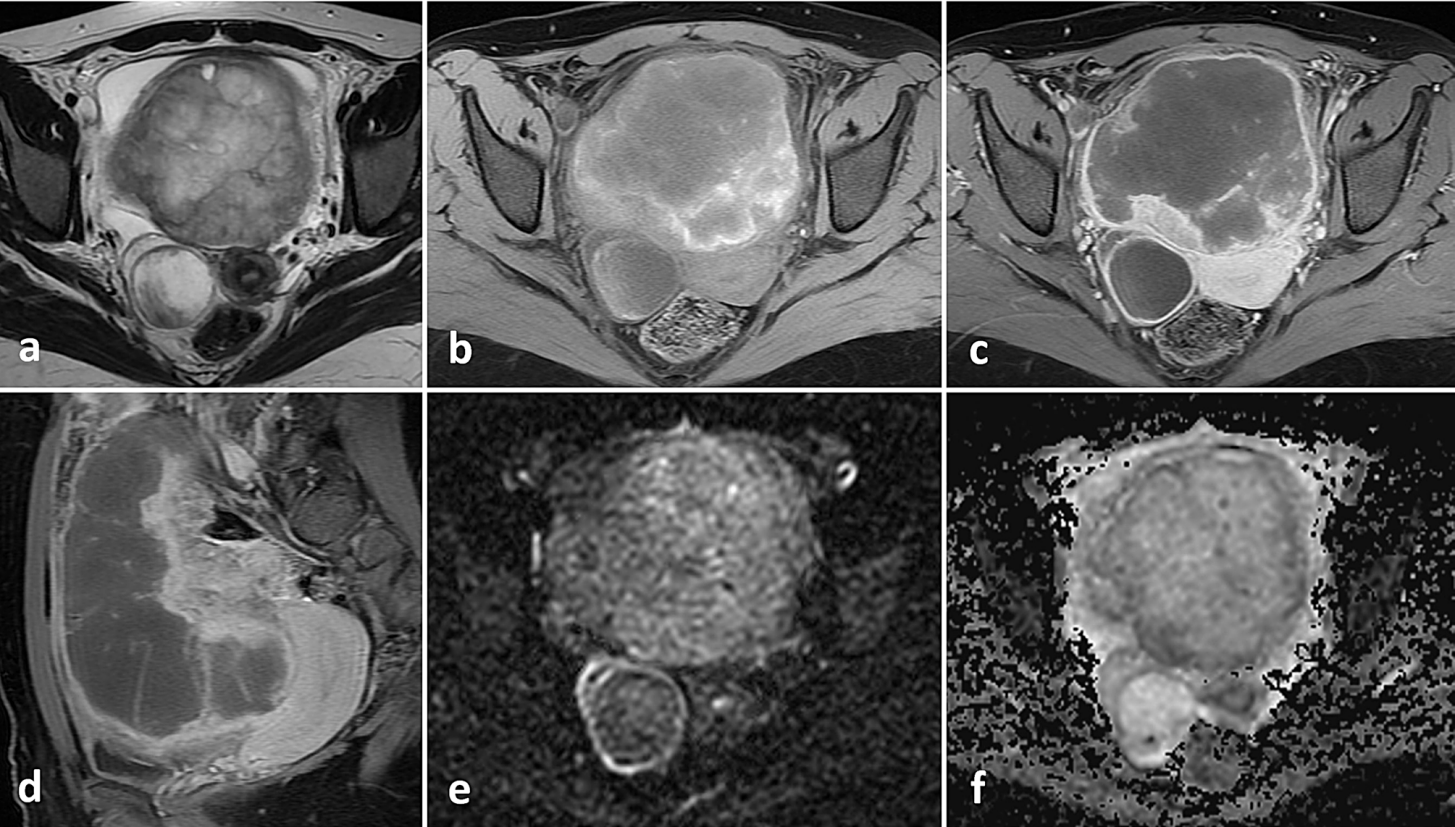
A left ovarian lesion of O-RADS MRI score 4, and a right ovarian lesion of O-RADS MRI score 3: (**a**) Axial T2-WI reveals a left-sided large multilobulated solid adnexal mass of intermediate signal intensity and irregular hypointense septa and a right ovarian cyst of mixed signal intensity. (**b**) Axial T1 fat-saturated image shows hypointensity of both lesions with few hyperintense areas denoting hemorrhagic changes. (**c** and **d**) Axial and sagittal T1 contrast-enhanced image; the left ovarian mass displays solid tissue and interlobular septal enhancement equal to the myometrium at 30–40 s, while the right ovarian cyst shows a smooth wall and septal enhancement with no detected enhancing solid tissue. (**e**) Axial DWI at b = 1000 revealed an intermediate signal of the left ovarian enhancing solid tissue and hypointensity at corresponding ADC image (**f**) denoting restricted diffusion pattern with an ADCmean value of 1.16 × 10 − 3 mm2 /s, the right ovarian cyst also revealed free diffusion pattern (apart from a small hemorrhagic area) with an ADCmean value of 2.02 × 10 − 3 mm2 /s. Pathology revealed left ovarian dysgerminoma and right ovarian benign hemorrhagic cyst

**Fig. 4:**
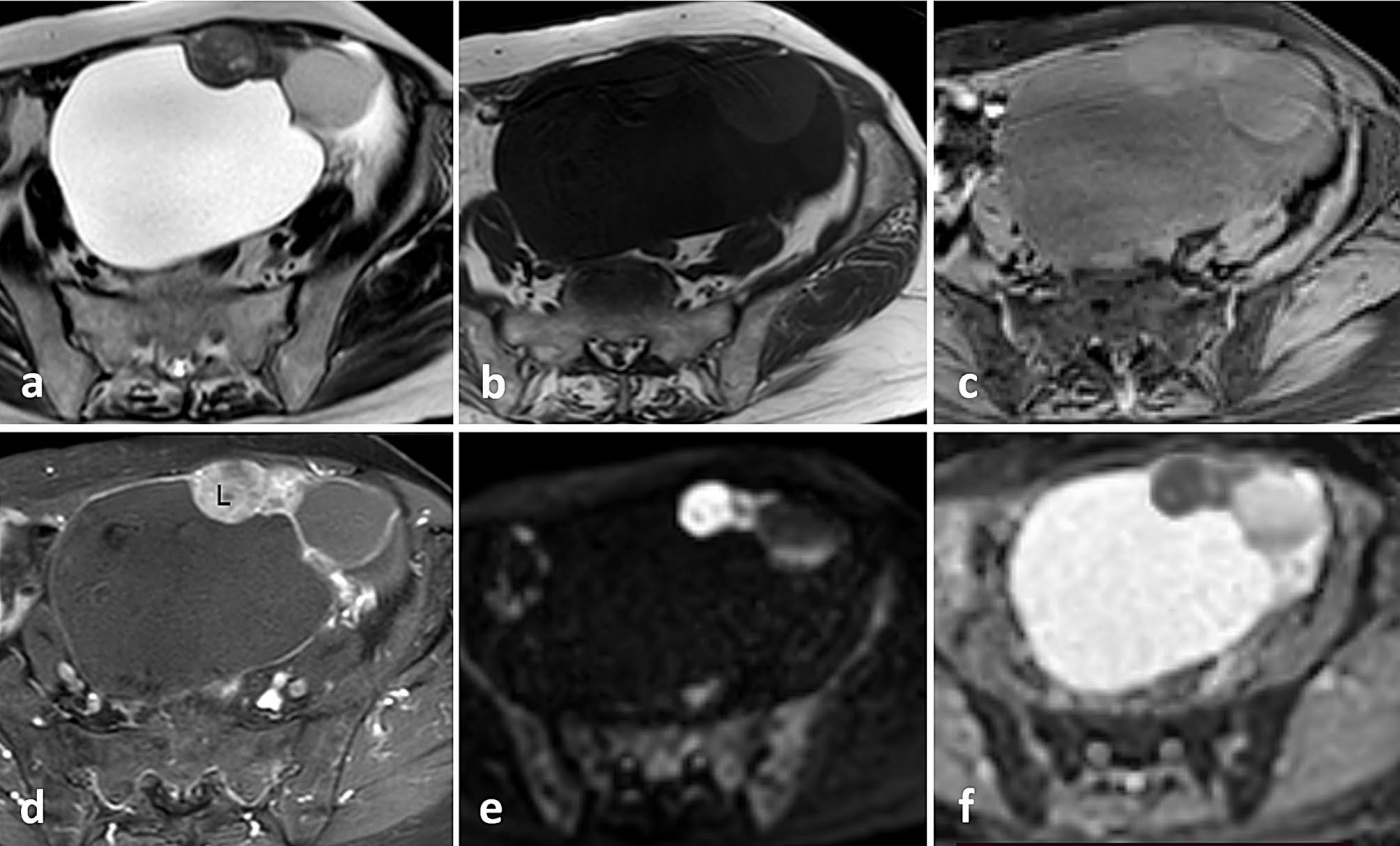
A left ovarian O-RADS MRI score 5 lesion. (**a**) Axial T2-WI reveals midline multilocular cystic adnexal mass with a large solid tissue of intermediate signal intensity. (**b**) Axial T1-WI revealed intermediate signal intensity locule without a signal drop on T1 fat-saturated image (**c**), denoting hemorrhagic content (**d**) Axial T1 contrast-enhanced images reveal avidly enhancing solid lesion (L) more than the myometrium (M) at 30–40 s. (**e**) Axial DWI at b = 1000 reveals a high signal of the solid tissue and hypointensity at the corresponding ADC image, denoting a restricted diffusion pattern. Pathology revealed ovarian high-grade serous carcinoma

No malignant lesions were reported as O-RADS MRI score 1 or 2. We reported only two malignant lesions of O-RADS-MRI score 3; they were diagnosed as metastatic deposits of low-grade appendicular mucinous neoplasm. We reported three benign lesions of O-RADS-MRI score 4: two fibrothecoma with edematous changes and one degenerated subserous myoma. No benign lesions with an O-RADS-MRI score of 5 were reported. Demonstrative cases are shown in Figs. 2, 3 and 4 and supplementary Figs. 2–5.

#### Qualitative DWI analysis

The high DW signal of the solid component at b1000 with corresponding ADC hypointensity and low DW signal of the cystic component was statistically significantly higher in malignant lesions than in benign lesions (*p* < 0.001).(Table 2).

#### Quantitative DWI analysis

##### ADCmean of the solid component

**Fig. 5:**
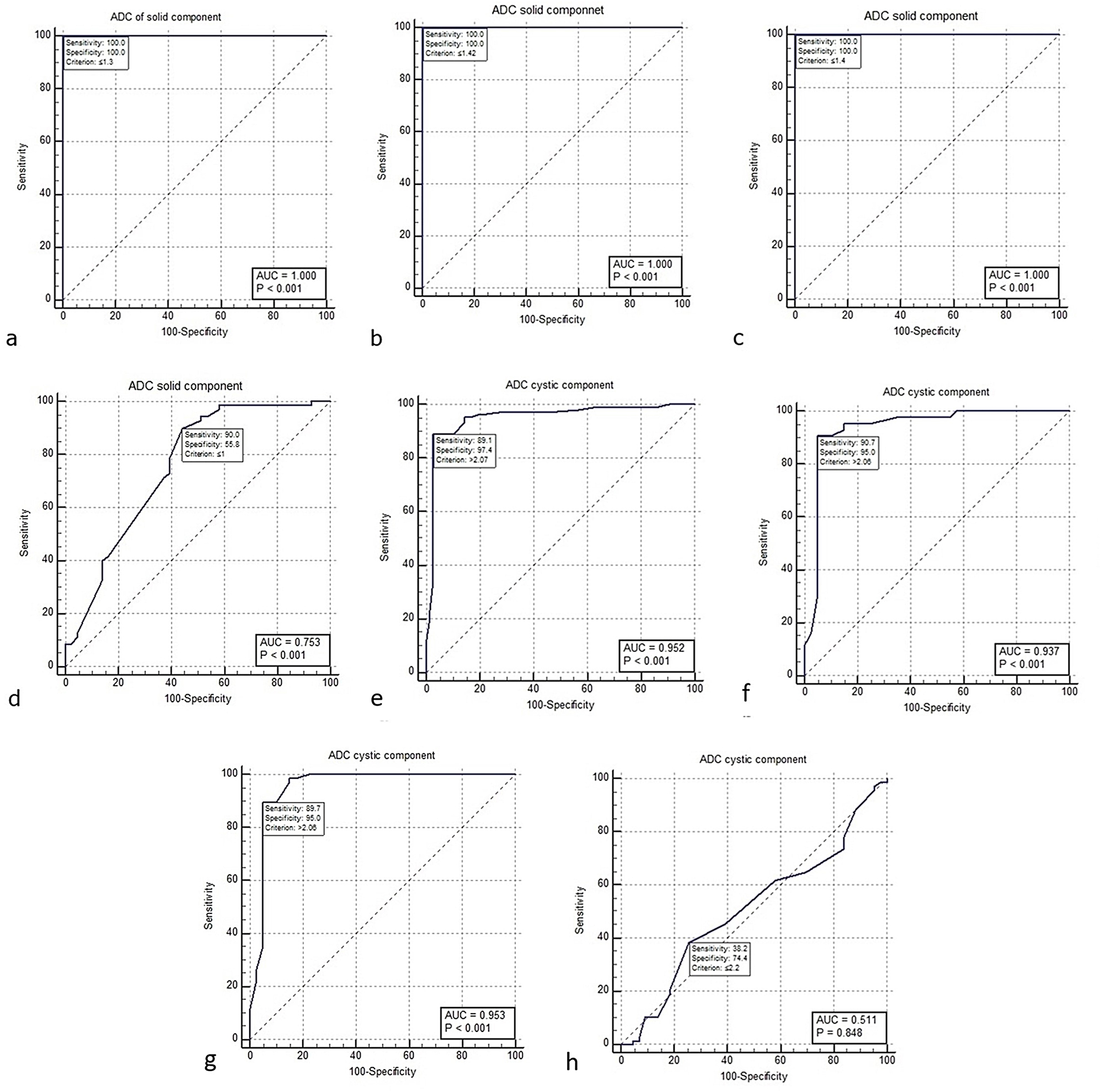
(**A**-**D**) ROC curves for diagnostic performance of ADCmean of solid component, to discriminate malignant from benign adnexal masses (**A**), O-RADS MRI score 4 vs. O-RADS MRI score 3 (**B**), O-RADS MRI score 5 vs. O-RADS MRI score 3 (**C**), and O-RADS MRI score 5 vs. O-RADS MRI score 4 (**D**). (**E**-**H**) ROC curves for diagnostic performance of ADCmean of cystic component, to discriminate malignant from benign adnexal masses (**E**), O-RADS MRI score 4 vs. O-RADS MRI score 3 (**F**), O-RADS MRI score 5 vs. O-RADS MRI score 3 (**G**), and O-RADS MRI score 5 vs. O-RADS MRI score 4 (**H**)

The ROC curve analysis revealed that ADCmean of the solid component was statistically significantly lower in malignant lesions (AUC = 1.000, *p* < 0.001). ADCmean, at a cut-off value of ≤ 1.3mm2/s, was a perfect discriminator between malignant and benign lesions with AUC = 1.000, with 95% CI: 100%–100%, sensitivity 100%,specificity100%, true positive rate (TPR) 100%, and false positive rate (FPR) 0%. ADCmean of the solid component at cut-off values of ≤ 1.42mm2/s and ≤ 1.4mm2/s were excellent discriminators for differentiating O-RADS 4 vs. O-RADS 3 and O-RADS 5 vs. O-RADS 3(AUC = 1.000 and 1.000; 95% CI: 100%–100% and 100%–100%, sensitivity 100% and 100%, specificity 100% and 100%, respectively). While a cut-off value of ≤1mm2/s revealed fair diagnostic accuracy for differentiation O-RADS 5 vs. O-RADS 4 (AUC = 0.753, 95% CI: 66%–85%), sensitivity 90%, specificity 55.8%, TPR 90%. FPR 44.2% (Fig. 5).

##### ADCmean of the cystic component

The ROC curve analysis revealed that ADCmean of the cystic component was statistically significantly higher in malignant lesions (AUC = 0.952, *p* < 0.001). ADCmean, at a cut-off value of > 2.07 × 10 –3 mm²/s, was an excellent discriminator for differentiating malignant and benign lesions with 89.1% and 97.4% sensitivity and specificity, respectively. ADCmean of the cystic component at a cut-off value of > 2.06mm2/s was an excellent discriminator for differentiating O-RADS 4 vs. O-RADS 3 and O-RADS 5 vs. O-RADS 3 (AUC = 0.937 and 0.953; sensitivity 90.7% and 89.7%; specificity 95% and 95% respectively). The ADCmean of the cystic component could not accurately differentiate between O-RADS 5 and O-RADS 4 (*p* = 0.848).

The PLR for ADCmean of the cystic component was 23. As an explanatory example, this means that a pretest probability of only 10% will be revised to a posttest probability of 72% in lesions with ADC cystic > 2.07 × 10 –3 mm²/s. The negative likelihood ratio (NLR) for ADCmean of the cystic component was 0.128. As an explanatory example, this means that a pretest probability of 50% will be revised to a posttest probability of only 12% in lesions with ADC cystic < 2.07 × 10 –3 mm²/s.

The ADCmean were compared between the O-RADS categories to explore the correlation between them and to investigate if considering ADC in O-RADS MRI can affect the management strategy later. There was a statistically significant difference in ADCmean values (of both solid and cystic components) between malignant vs. borderline tumors and malignant vs. benign tumors, but not between borderline vs. benign tumors **(**Table 5**)**.

**Table 5:** ADC of solid and cystic components in different pathological types

ADC	Pathology			H (2)	*P* value	P1	P2	P3
	Benign	Borderline	Malignant					
Solid	1.44(1.41–1.54)	1.21(1.2–1.22)	0.9(0.8–0.99)	52.56	**< 0.001**	1	< 0.001	< 0.001
Cystic	1.72(1.3–1.85)	2.45(2.38–2.53)	2.4(2.2–2.5)	112.38	**< 0.001**	0.702	< 0.001	< 0.001

Data is median (Q1-Q3). The test of significance is Kruskal-Wallis H-test. Pairwise comparisons with Bonferroni correction for multiple tests: P1: Benign vs. borderline, P2: benign vs. malignant, and P3: borderline vs. malignant

### Misclassified lesions

There were two malignant lesions of O-RADS-MRI score 3 without solid tissue; they were diagnosed as metastatic deposits of low-grade appendicular mucinous neoplasm. While three benign lesions of O-RADS-MRI score 4: two fibrothecoma with edematous and cystic changes, and one degenerated subserous myoma. The ADCmean values may have additional information to O-RADS, helping accurately diagnose challenging adnexal lesions with a misclassified O-RADS MRI score. The two malignant lesions of misclassified O-RADS-MRI score 3 revealed ADCmean of cystic component > 2.07 × 10 –3 mm²/s. While the three benign lesions of misclassified O-RADS-MRI score 4 revealed ADCmean of solid and cystic components > 1.3 × 10 –3 mm²/s and ≤ 2.07 × 10 –3 mm²/s respectively.

### Inter-rater agreement

There was excellent agreement between the two readers in assessing O-RADS categories (k_w_=0.901 and 95% CI = 0.863–0.939). Both readers agreed upon all five O-RADS 1 lesions. Both agreed on thirty-one O-RADS 2 lesions and disagreed on two as the first reader diagnosed them as O-RADS 3. Both readers agreed on thirty-six O-RADS 3 lesions; the first reader diagnosed one case as O-RADS 2. Regarding O-RADS 4 lesions, both readers agreed on twenty-nine lesions and disagreed on eight lesions; three were diagnosed as O-RADS 3, while five were diagnosed as O-RADS 5 by the first reader. Regarding O-RADS 5 lesions, both readers agreed on sixty-five lesions and disagreed on fourteen lesions, which the first reader diagnosed as O-RADS 4. Disagreement occurred only within one higher or lower O-RADS category (Table 6).

**Table 6:** Inter-rater reliability in assessing O-RADS categories

Observer 2	Observer 1						Weighted Kappa			
	**O-RADS 1**	**O-RADS 2**	**O-RADS 3**	**O-RADS 4**	**O-RADS 5**	**Total**	**K**_**w**_	**95%CI**		**SE**
O-RADS 1	5	0	0	0	0	5 (2.6%)	0.901	0.863 to 0.939	0.019	
O-RADS 2	0	31	2	0	0	33 (17.3%)				
O-RADS 3	0	1	36	0	0	37 (19.4%)				
O-RADS 4	0	0	3	29	5	37 (19.4%)				
O-RADS 5	0	0	0	14	65	79 (41.4%)				
Total	5 (2.6%)	32 (16.8%)	41 (21.5%)	43 (22.5%)	70 (36.6%)	191				

**Fig. 6:**
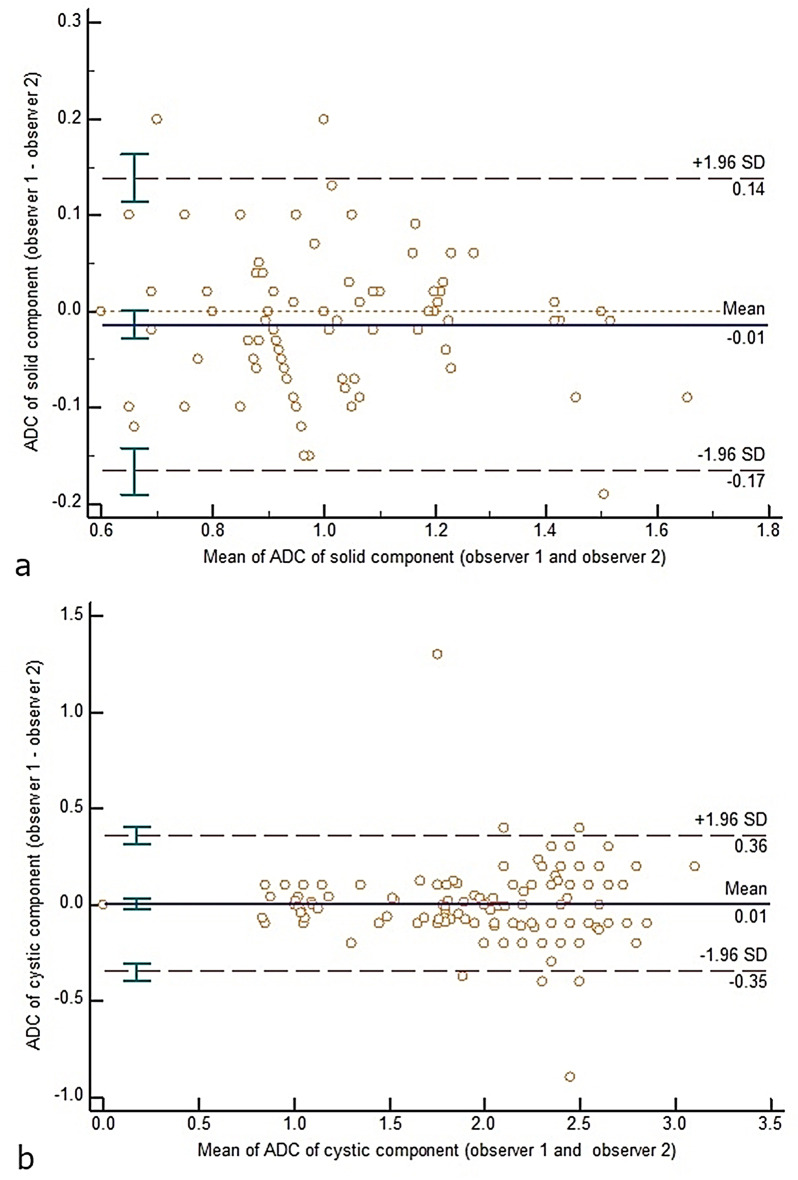
Bland-Altman plots for inter-rater reliability of ADCmean measurements for solid (**A**) and cystic (**B**) components

Each reader measured three hundred five ADCmean values. There was excellent reliability (absolute agreement) between the two readers (*p* < 0.001) in measuring ADC values for both solid and cystic components of adnexal masses with ICC = 0.992 and 95% CI = 0.990–0.994, ICC = 0.932 and 95% CI = 0.910–0.949, respectively (Fig. 6).

### Predictors for malignancy

The binary logistic regression analysis was performed to determine the effects of lesion size, laterality, presence of simple fluid, endometriotic fluid, lipid-containing fluid, DW signal, and ADC mean value of the cystic component on the likelihood of ovarian masses malignancy. The ADCmean of solid component was not included in the logistic regression as it was a perfect discriminator for differentiating malignant and benign lesions with 100% and 100% sensitivity and specificity, respectively. This exclusion was a mathematical requirement of standard Maximum Likelihood Estimation (MLE) logistic regression. On univariate analysis, lesion size, presence of endometriotic fluid, DW signal of the cystic component, and ADC value of the cystic component were statistically significant predictors of malignancy. Then a multivariate logistic regression model with backward elimination was performed. In step 4, the model variables entered were lesion size, presence of simple fluid, DW signal of cystic component, and ADC of the cystic component. The model was statistically significant c2(4) = 175.140, p value < 0.001. The model correctly classified 94.5% of cases with a sensitivity and a specificity of 92.5% and 97.4%, respectively. Among the four predictor variables, the presence of simple fluid, low DW signal of cystic component, and ADC of cystic component > 2.07 × 10 –3 mm²/s were statistically significant independent predictors of malignancy. An adnexal lesion of O-RADS MRI score > 3 with ADCmean of cystic component > 2.07 × 10 –3 mm²/s has 221 times higher odds of exhibiting malignancy (Table 7).

**Table 7:** Logistic regression analysis for predictors of malignancy

Predictor	Univariable			Multivariable		
	HR	95% CI	*p*-value	HR	95% CI	*p*-value
Lesion size (cm)			**0.011**			0.055
≤5	r(1)	r(1)		r(1)	r(1)	
>5	2.5	1.2–4.2		4.1	0.97–17.5	
Laterality			**< 0.001**			
Unilateral	r(1)	r(1)		-	-	
Bilateral	59	3-11.3				
Simple fluid			0.103			**0.008**
Absent	r(1)	r(1)		r(1)	r(1)	
Present	1.9	0.9–3.9		27.2	2.3-316.4	
Endometriotic			**0.002**			
Present	r(1)	r(1)		-	-	
Absent	23.9	3-186				
Lipid containing			0.072			
Present	r(1)	r(1)		-	-	
Absent	7.3	0.8–64				
DW signal of cystic component			**< 0.001**			**0.008**
High	r(1)	r(1)		r(1)	r(1)	
Low	54.5	12.5–237		47.8	3.3–683	
ADC cystic			**< 0.001**			**< 0.001**
≤ 2.07	r(1)	r(1)		r(1)	r(1)	
>2.07	149	46.2–481		221	41.2–1138	

r(1), reference category; HR, hazard ratio; CI, confidence interval

## Discussion

To the best of our knowledge, this is the first study to assess the diagnostic reliability of O-RADS-MRI score based on non-DCE-MRI protocol and ADC measurements. Our results revealed excellent inter-rater reliability in assessing O-RADS-MRI score with a statistically significant higher malignancy rate in masses with O-RADS-MRI > 3. That was in line with the recent prospective study by Pereira et al. to assess the performance of O-RADS-MRI score utilizing DCE-MRI and another retrospective study by Aslan et al. using O-RADS-MRI score based on simplified DCE-MRI [11, 23]. Unlike our results, neither inter-rater reliability nor correlation with ADC values was performed in both studies. Furthermore, this current study revealed high PPV for O-RADS 4–5 (97.3%) with comparable diagnostic performance to Pereira et al. study that utilized O-RADS-MRI Based on DCE-MRI [23]. Similarly, a recent retrospective study (not utilizing O-RADS-MRI) revealed comparable diagnostic accuracy of non-DCE MRI (81–88%) as compared to DCE-MRI (81–85%) for distinguishing malignant and benign adnexal masses [24]. Nevertheless, another DCE-MRI-based retrospective study revealed that time-intensity curves analysis was more accurate than visual assessment for accomplishing ideal diagnostic accuracy with the O-RADS-MRI score [25]. These results may be attributed to a larger number of included patients in their study. We reported a high prevalence of malignancy (112/191 lesions, 59%) because our hospital is a tertiary care center with malignancy rates of 0%, 0%, 4.8%, 93%, and 100% for O-RADS-MRI scores of 1,2,3,4, and 5, respectively. Our results were in a concordance study conducted by Aslan et al., except for their lower malignancy rate of O-RADS-MRI score of 4 (50%). This difference is assumed to be the lower number of malignant lesions in their study [11].

None of the lesions with O-RADS-MRI scores of 1 and 2 in the current study revealed malignant pathology or signs of malignancy in the follow-up imaging, which was in line with previous studies utilizing the ADNEX-MR score [9, 26] and O-RADS-MRI score [11, 23]. Therefore, lesions with O-RADS-MRI score 1–2 can be followed up. In this study, among 41 lesions with an O-RADS-MRI score 3, only two were malignant without solid tissue (low-grade appendicular mucinous neoplasm). Our results were similar to previous studies utilizing the DCE-MRI protocol [11, 23]. The assessment of multiloculated O-RADS 3 lesions with thick septation without solid tissue is challenging, and therefore, signal analysis of the cystic component is advised [26]. We recommend that lesions with an O-RADS 3 can undergo minimally invasive surgery or be followed meticulously.

O-RADS-MRI score 4 lesions show solid tissue with enhancement less than the myometrium. Among forty-three O-RADS-MRI score 4 lesions in this study, 40 had malignant pathology. Misclassified lesions were due to misinterpretation of a solid component exhibiting non-homogenous dark T2/ dark DWI signal or incorrect localization of mass origin, which was in line with interpretive causes reported in recent studies [23, 27]. However, adding ADC measurements to O-RADS-MRI score 4 is recommended to reduce the false-positive rate as lower ADC values of solid tissue of malignant masses are noted. Additionally, correlation with tumor markers and reporting the related possibilities of malignancy may help the gynecologic oncologist to plan the best surgical alternative to avoid unnecessary extensive surgery. O-RADS-MRI score 5 lesions show solid tissue with enhancement more than the myometrium or lesions associated with peritoneal implants. All included O-RADS 5 lesions were malignant, which was in concordance with previous studies [11, 23]. Our results revealed no borderline tumors of O-RADS 5, as in previous studies, which revealed a very low probability of a borderline tumor when a lesion scores 5 [1, 9]. In patients with an O-RADS-MRI score of 4 or 5, referral to a gyne-oncologist may be considered. In our analysis, borderline ovarian tumors (*n* = 14) were classified alongside malignancies to align with clinical triage protocols, as both require specialized surgical evaluation and borderline ovarian tumors cannot be managed conservatively like several benign lesions. From an imaging perspective, borderline tumors typically exhibit intermediate cellular density without true stromal invasion, resulting in ADC values that fall in a mid-range between benign lesions and malignancy.

Previous meta-analysis (not utilizing O-RADS-MRI) reported ADC values of solid components distinguishing malignant from benign adnexal masses varying from 1.15 to 1.25 × 10^-3^mm^2^/sec [28]. However, another meta-analysis emphasized the difficulty of establishing a malignant ADC cut-off value in ovarian cancer [29]. Our results revealed that the high DW signal intensity of the solid component at b1000 with corresponding ADC hypointensity was statistically significantly higher in malignant lesions, which was in line with a study using the ADNEX-MR score [30]. ADCmean values of solid tissue in malignant lesions were significantly lower than those of benign lesions, which concord with a study that assessed the value of quantitative DWI in O-RADS-MRI score [31]. We found that an ADCmean of the solid component ≤ 1.3 × 10^-3^mm^2^/s is an excellent discriminator to predict malignancy. Similarly, a recent study considered an ADC mean value ≤ 1.08 × 10^-3^mm^2^/s as the optimal threshold to predict malignant adnexal lesions [32].

The ADC analysis of the cystic component has been proven to be valuable in discriminating between benign and malignant intracranial, hepatic, and pancreatic tumors [33–35]. Few studies investigated ADC analysis of the cystic component in complex ovarian lesions [14]. The malignant ovarian masses revealed free diffusion and high ADC values of the cystic component, which can be rationalized by the necrotic, non-viable or molecular component of the serous parts. In contrast, the pelvic abscess revealed low ADC values due to the high viscosity of the pus cells, which markedly limits the water proton motion.

The low DW signal of the cystic component in the current study was significantly higher in malignant lesions with an ADCmean of the cystic component > 2.07 × 10 –3 mm²/s as an independent predictor of malignancy. Furthermore, a cut-off value of > 2.06mm2/s was an excellent discriminator for differentiating O-RADS 4 vs. O-RADS 3 and O-RADS 5 vs. O-RADS 3. That was in agreement with Assouline et al., who concluded that DWI analysis of the cystic component enhanced the diagnosis performance of the O-RADS score and reported that ADCmean of the cystic component > 1.69 × 10-3mm2/s is associated with malignancy [36].

The drawback of using non-DCE-MRI to assess the O-RADS-MRI score is the inability to differentiate between O-RADS 3 and 4 lesions with enhancing soft tissue components (less than myometrium) as non-DCE-MRI does not allow for TIC generation. Nevertheless, O-RADS-MRI score 3 lesions in the current study were unilocular cystic lesions with non-simple non-endometriotic fluid content, multilocular cystic lesions or hematosalpinx with no solid component.

This study has a few limitations. Firstly, the single-center retrospective study design. Future prospective, multi-center trials including consecutive patient recruitment, utilizing diverse scanner types, and reader experience levels are crucial for assessing the impact on clinical management and ensuring broader applicability in clinical settings. Secondly, the small number of included borderline tumors (< 8%), further larger specific studies are recommended. Thirdly, the lack of comparison between DCE-MRI and non-DCE MRI, further comparative studies may add valuable data. Fourthly, our results revealed a statistically significant difference and absolute inter-rater agreement in measuring ADC values for both solid and cystic components of adnexal masses. The ADCmean of the solid component revealed AUC = 1.000 with the absence of external validation or multicenter data for the proposed ADC cut-offs. We recommend further larger studies to validate the ADC cut-off values. The variability of ADC between machines and b values should be considered. Fifthly, the high malignancy prevalence may limit the generalizability of PPV/NPV and management recommendations to lower-risk populations.

## Conclusion

Using the O-RADS-MRI score based on non-DCE-MRI resulted in a diagnostic performance comparable to previous studies that used DCE-MRI, which could facilitate the widespread application of the O-RADS-MRI score. Non-DCE-MRI will save time compared to DCE-MRI, which will be of great significance in patients who cannot stand the extended examination time of DCE-MRI or have marked ascites commonly seen in the malignant lesions with peritoneal involvement.

Based on our results, ADC provides an applicable rapid quantitative predictor of malignancy, enhancing the O-RADS-MRI score risk stratification of adnexal masses and consequently improving patient outcomes. Incorporating the DWI quantitative analysis in the O-RADS-MRI score may be crucial for determining a risk stratification with consequent clinical management, especially in a group without dynamic perfusion data, such as renal failure and pregnancy cases.

## Supplementary Information

Below is the link to the electronic supplementary material.

Supplementary Material 1: Supplementary figure (1): Malignant and benign lesions solid tissue enhancement at non-DCE MRI.

Supplementary Material 2: Supplementary figure (2): A left ovarian lesion of O-RADS MRI score 4. (a&b) Axial & sagittal T2-WI reveal a left-sided complex cystic adnexal mass with multiple solid tissue of intermediate signal intensity and abnormal endometrial thickening. (c& d) Axial and sagittal T1 contrast-enhanced image; the left ovarian mass reveals solid tissue enhancement less than and equal to the myometrium at 30-40 sec, while the endometrial thickening reveals heterogenous enhancement. (e) Axial DWI at b=1000 reveals a high signal of the solid tissue and hypointensity at corresponding ADC image (f), denoting a restricted diffusion pattern. Pathology revealed synchronous left ovarian endometroid carcinoma and endometrial endometroid carcinoma.

Supplementary Material 3: Supplementary figure (3): Bilateral ovarian lesions of O-RADS MRI score 5 lesion. (a) Axial T2-WI reveals bilateral complex adnexal masses with a predominant solid tissue of intermediate signal intensity and moderate ascites. (b) Axial T1-WI image shows hypointensity of both masses. (c and d) Axial, and sagittal T1 contrast-enhanced image reveals avidly enhancing solid tissue more than the myometrium at 30-40 sec. (e) Axial DWI at b=1000 reveals a high signal of the solid tissue and hypointensity at corresponding ADC image (f), denoting a restricted diffusion pattern. Pathology revealed bilateral ovarian serous carcinoma.

Supplementary Material 4: Supplementary figure (4): A left ovarian lesion of O-RADS MRI score 4. (a) Axial T2-WI reveal a left-sided solid adnexal mass of intermediate to low signal intensity with cystic changes and moderate ascites. (b) Axial T1-WI image shows hypointensity of the mass (c& d) Axial and sagittal T1 contrast-enhanced image; the left ovarian mass reveals faint enhancement less than the myometrium at 30-40 sec. (e) Axial DWI at b=1000 reveals few foci of intermediate signal of the solid tissue and hypointensity at corresponding ADC image (f), denoting a mild restricted diffusion pattern. Pathology revealed left ovarian fibrothecoma with cystic changes and edema.

Supplementary Material 5: Supplementary figure (5): A right ovarian lesion of O-RADS MRI score 3. (a) Axial & coronal T2-WI reveal a right-sided multilocular cystic adnexal mass with thin septae. (c) Axial T1-WI image shows hypointensity of the lesion. (d) Axial T1 contrast-enhanced image reveals smooth septal and wall enhancement, no enhancing solid tissue. (e) Axial DWI at b=1000 reveals a low signal of the lesion and hyperintensity at corresponding ADC image (f), denoting a free diffusion pattern. Pathology revealed right ovarian mucinous cystadenoma.

Supplementary Material 6: Supplementary Table (1): The O-RADS distribution and corresponding malignancy rates

## Data Availability

All data generated or analyzed during this study are included in this published article.
