# Supplementary figures and images for: Diagnostic reliability of O-RADS score based on non-dynamic contrast-enhanced MRI and apparent diffusion coefficient in characterization of adnexal masses
Source: BMC Med Imaging. 2026 Jun 23;26:310. doi: 10.1186/s12880-026-02498-7 (PMC13292438; doi:10.1186/s12880-026-02498-7)

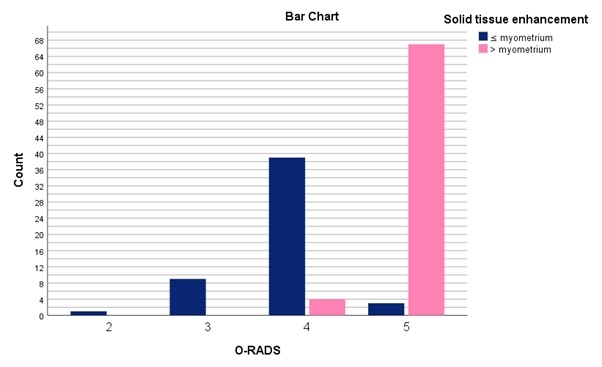

Supplement: Supplementary file 1 — Supplementary Material 1: Supplementary figure (1): Malignant and benign lesions solid tissue enhancement at non-DCE MRI. [file 12880_2026_2498_MOESM1_ESM.jpg]

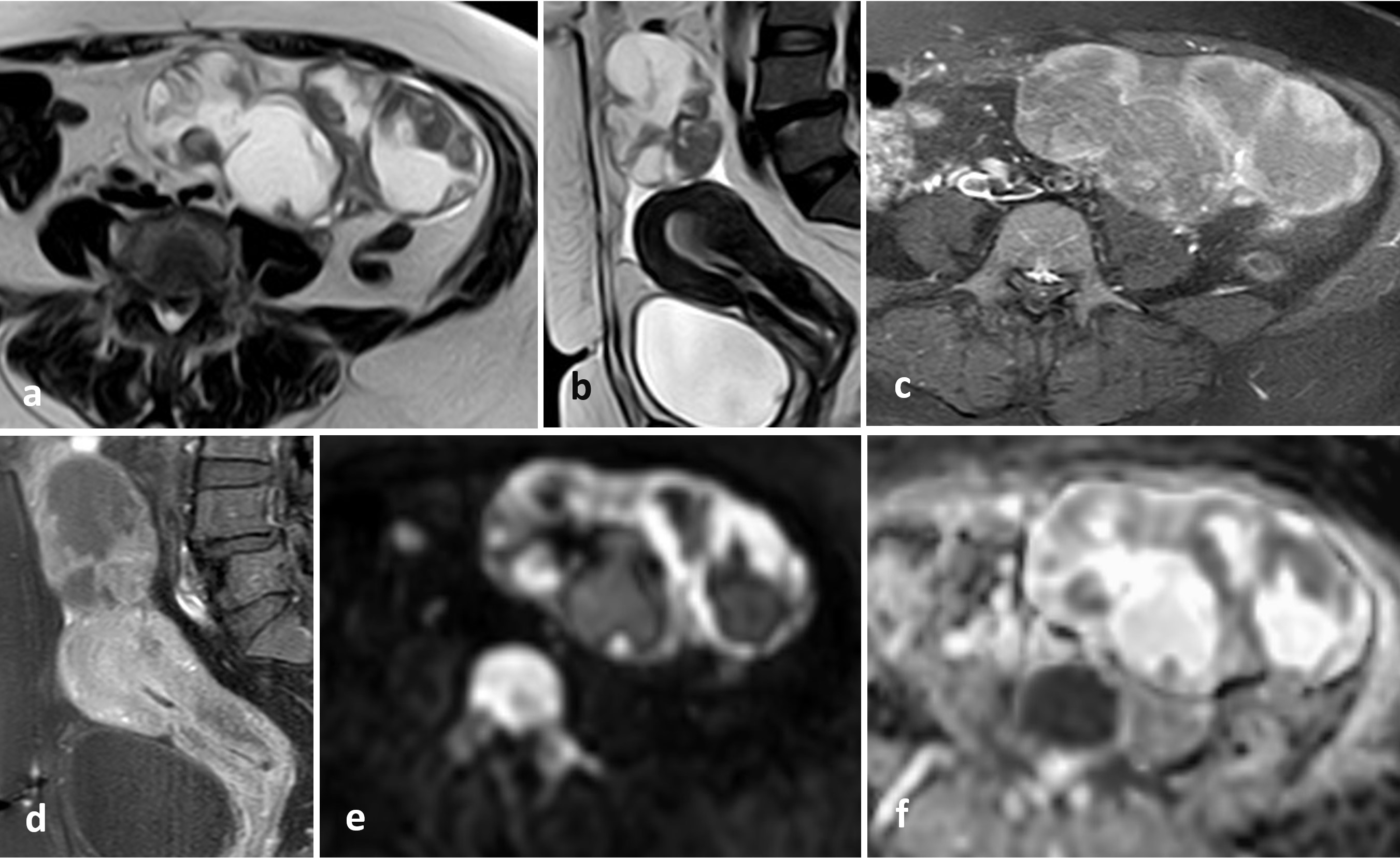

Supplement: Supplementary file 2 — Supplementary Material 2: Supplementary figure (2): A left ovarian lesion of O-RADS MRI score 4. (a&b) Axial & sagittal T2-WI reveal a left-sided complex cystic adnexal mass with multiple solid tissue of intermediate signal intensity and abnormal endometrial thickening. (c& d) Axial and sagittal T1 contrast-enhanced image; the left ovarian mass reveals solid tissue enhancement less than and equal to the myometrium at 30-40 sec, while the endometrial thickening reveals heterogenous enhancement. (e) Axial DWI at b=1000 reveals a high signal of the solid tissue and hypointensity at corresponding ADC image (f), denoting a restricted diffusion pattern. Pathology revealed synchronous left ovarian endometroid carcinoma and endometrial endometroid carcinoma. [file 12880_2026_2498_MOESM2_ESM.jpg]

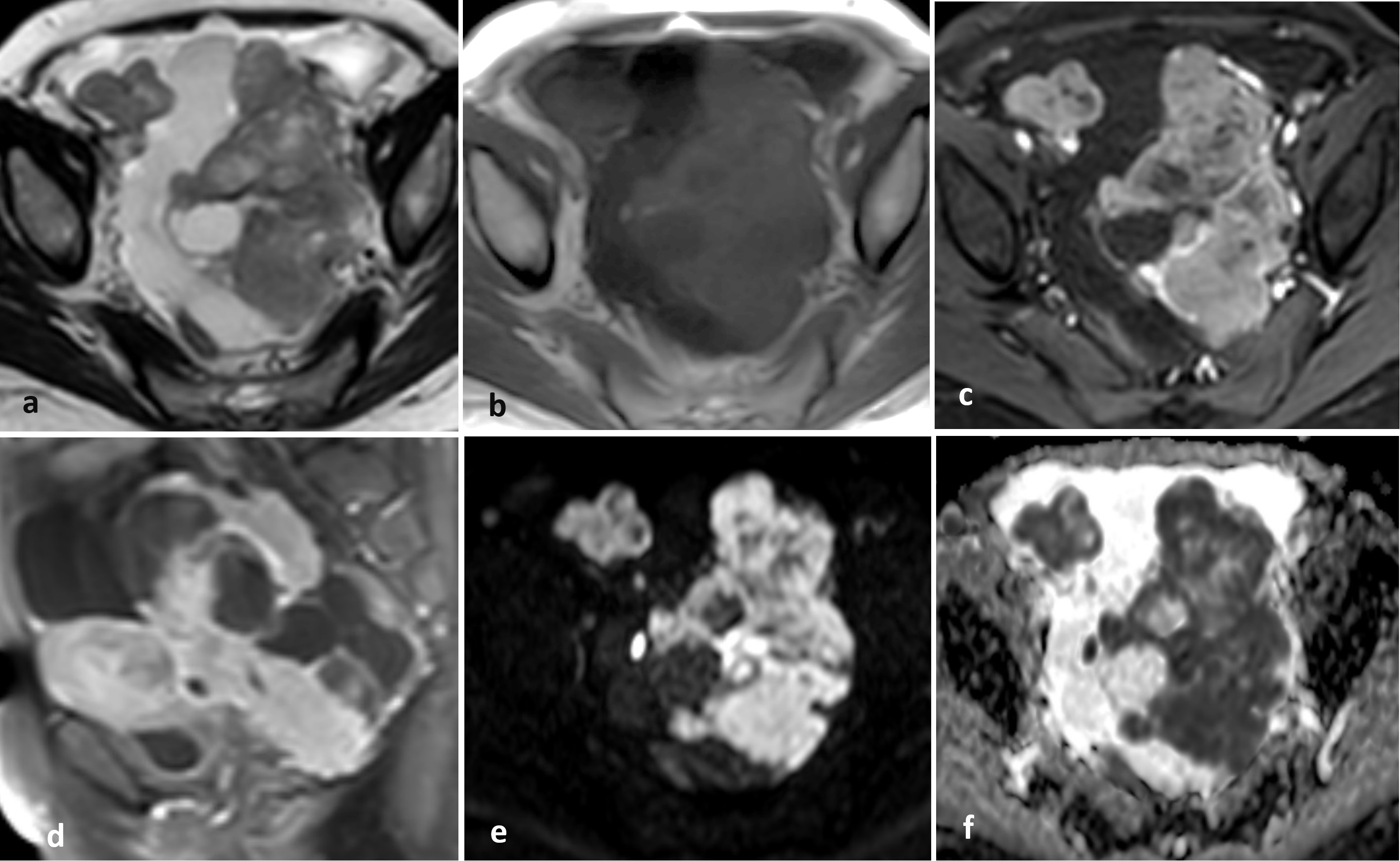

Supplement: Supplementary file 3 — Supplementary Material 3: Supplementary figure (3): Bilateral ovarian lesions of O-RADS MRI score 5 lesion. (a) Axial T2-WI reveals bilateral complex adnexal masses with a predominant solid tissue of intermediate signal intensity and moderate ascites. (b) Axial T1-WI image shows hypointensity of both masses. (c and d) Axial, and sagittal T1 contrast-enhanced image reveals avidly enhancing solid tissue more than the myometrium at 30-40 sec. (e) Axial DWI at b=1000 reveals a high signal of the solid tissue and hypointensity at corresponding ADC image (f), denoting a restricted diffusion pattern. Pathology revealed bilateral ovarian serous carcinoma. [file 12880_2026_2498_MOESM3_ESM.jpg]

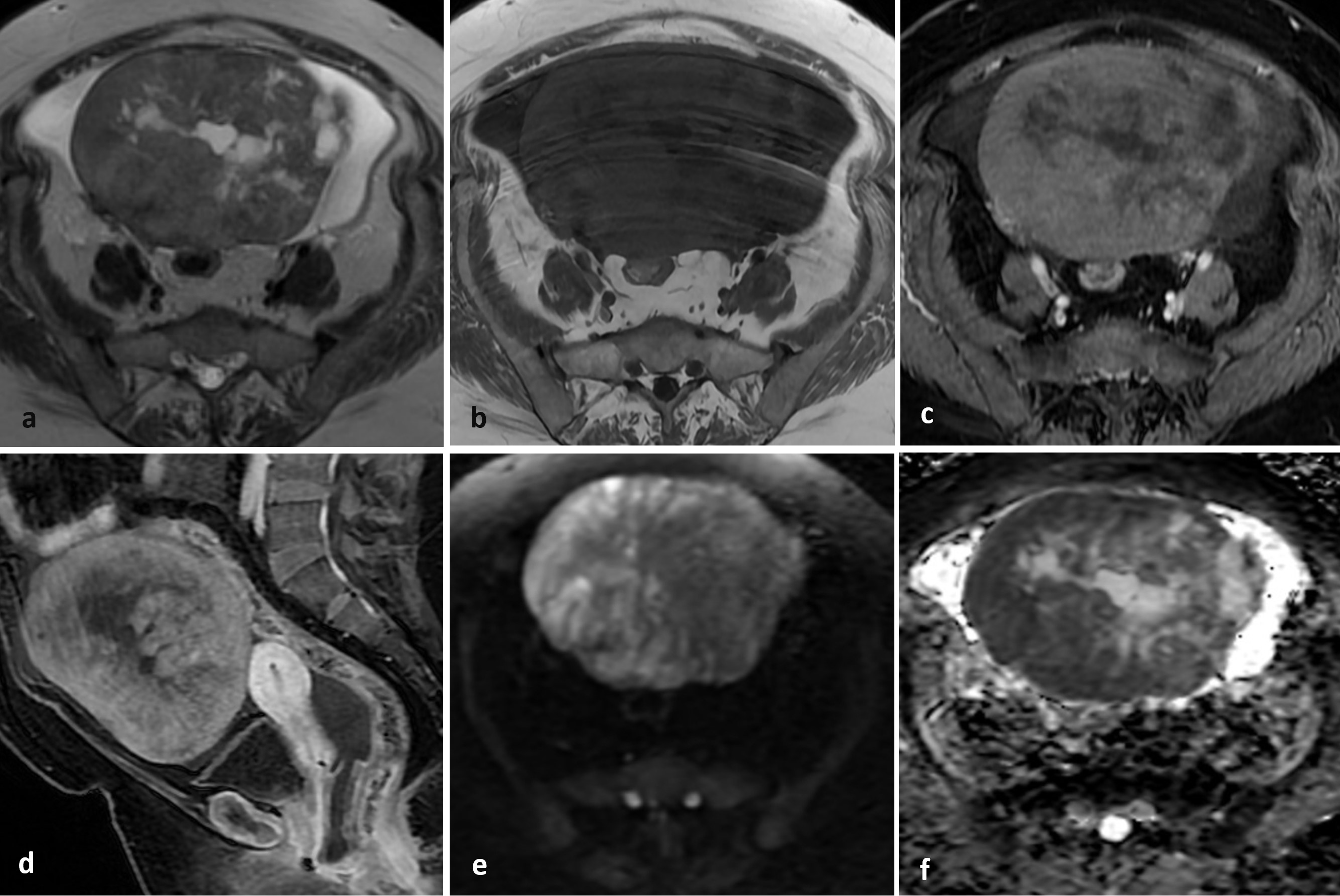

Supplement: Supplementary file 4 — Supplementary Material 4: Supplementary figure (4): A left ovarian lesion of O-RADS MRI score 4. (a) Axial T2-WI reveal a left-sided solid adnexal mass of intermediate to low signal intensity with cystic changes and moderate ascites. (b) Axial T1-WI image shows hypointensity of the mass (c& d) Axial and sagittal T1 contrast-enhanced image; the left ovarian mass reveals faint enhancement less than the myometrium at 30-40 sec. (e) Axial DWI at b=1000 reveals few foci of intermediate signal of the solid tissue and hypointensity at corresponding ADC image (f), denoting a mild restricted diffusion pattern. Pathology revealed left ovarian fibrothecoma with cystic changes and edema. [file 12880_2026_2498_MOESM4_ESM.jpg]

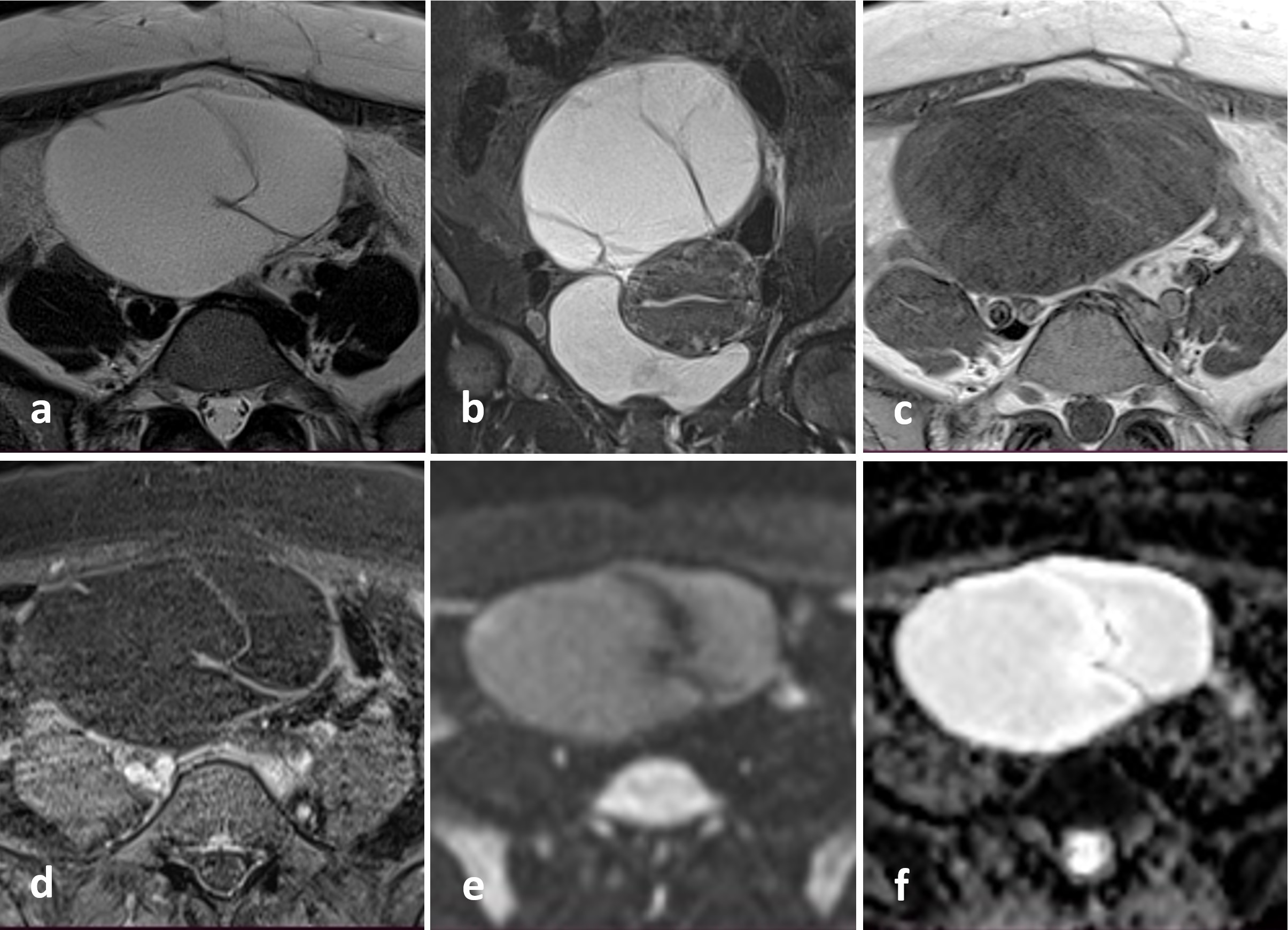

Supplement: Supplementary file 5 — Supplementary Material 5: Supplementary figure (5): A right ovarian lesion of O-RADS MRI score 3. (a) Axial & coronal T2-WI reveal a right-sided multilocular cystic adnexal mass with thin septae. (c) Axial T1-WI image shows hypointensity of the lesion. (d) Axial T1 contrast-enhanced image reveals smooth septal and wall enhancement, no enhancing solid tissue. (e) Axial DWI at b=1000 reveals a low signal of the lesion and hyperintensity at corresponding ADC image (f), denoting a free diffusion pattern. Pathology revealed right ovarian mucinous cystadenoma. [file 12880_2026_2498_MOESM5_ESM.jpg]
