# Supplementary material for: Diagnostic reliability of O-RADS score based on non-dynamic contrast-enhanced MRI and apparent diffusion coefficient in characterization of adnexal masses
Source: BMC Med Imaging. 2026 Jun 23;26:310. doi: 10.1186/s12880-026-02498-7 (PMC13292438; doi:10.1186/s12880-026-02498-7)
Supplement: Supplementary file 6 — Supplementary Material 6: Supplementary Table (1): The O-RADS distribution and corresponding malignancy rates [file 12880_2026_2498_MOESM6_ESM.docx]

**Supplementary Table (1): The O-RADS distribution and corresponding malignancy rates**

| O-RADS | Patient N. (%) | Malignancy rate |
| --- | --- | --- |
| O-RADS 1 | 5 (2.6 %) | 0 % |
| O-RADS 2 | 32 (16.8 %) | 0 % |
| O-RADS 3 | 41 (21.5%) | 4.9 % |
| O-RADS 4 | 43 (22.5%) | 93.0 % |
| O-RADS 5 | 70 (36.6%) | 100 % |
